# Hunt Trials as a Measure to Assess Level of Training in Boarhounds

**DOI:** 10.3390/ani11061661

**Published:** 2021-06-02

**Authors:** Elżbieta Bednarek, Anna Sławinska

**Affiliations:** Department of Animal Biotechnology and Genetics, UTP University of Science and Technology, Mazowiecka 28, 85-084 Bydgoszcz, Poland; elzbieta-bednarek2@wp.pl

**Keywords:** boarhound, terrier, dachshund, hound, scent (nose), tracking, attacking, obedience, courage, sharpness

## Abstract

**Simple Summary:**

Hunting dogs have been bred for centuries to assist people in their hunting activities. They possess excellent instincts and fitness. Evaluating the skills of hunting dogs is an important source of knowledge on how to use the hunting potential of dogs along with their training. Hunting trials consist of competitions that reflect different hunting situations. The results of the hunt trials of the boarhounds showed that there is a link between factors such as sex, age, breed group and breed and the performance of dogs. Boarhounds learn obedience easily, but they should practice other skills, such as tracking and announcing the game. Among different breed groups of boarhounds, the best performing ones were dachshunds.

**Abstract:**

Boarhounds are hunting dogs bred for hunting wild boar, including terriers, dachshunds, and hounds. Hunt trials evaluate the individual hunting potential and trainability of the boarhounds in ten different competitions. The aim of this study was to determine the factors influencing the hunt trials for boarhounds in a large cohort of hunting dogs. The analysis was conducted based on the results of hunt trials for boarhounds conducted in 2005–2015. The database contained 1867 individuals belonging to 39 breeds. Effects of sex, age, breed group, and breed were estimated by non-parametric analysis of variance. Sex influenced (*p* < 0.01) the total score, and in almost all competitions dogs performed better than bitches. Age affected (*p* < 0.01 or *p* < 0.05) all competitions, indicating that the dogs perform better with age. The results analyzed by the breed group showed that the dachshunds performed better in courage (*p* < 0.01) and searching (*p* < 0.05). Breed influenced (*p* < 0.01) almost all scores except obedience and tracking on the lead. The best performing breed was Alpine Dachsbracke. In conclusion, all analyzed factors influenced the results of the hunt trials. The factors with the largest impact were breed and age, which reflect both the hunting potential and the level of training of the boarhounds.

## 1. Introduction

**Figure 1:**
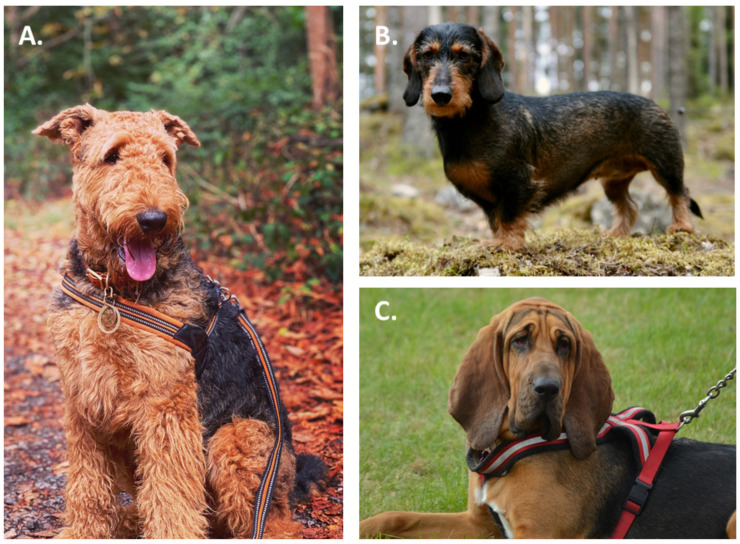
Typical representatives of boarhounds: (**A**) terrier: Welsh Terrier belongs to group III Section 1 large and medium sized terriers; (**B**) dachshund: Wire-haired Dachshund belongs to group IV dachshunds; (**C**) hounds: Bloodhound belongs to group VI Section 1 scent hounds.

Boarhounds are hunting dogs, specifically bred for hunting wild boar. Small boarhounds, such as terriers and dachshunds, are most often used for individual hunting. During individual hunting, the dog’s task is to find and surround the boar. On the other hand, large boarhounds, including hounds, are used in group hunting, which has different requirements from the dog. During group hunting, boarhounds are supposed to separate the wild boar from the herd, so that the hunter has a clear shot. There are different physical and psychological traits that boarhounds have to carry to be able to effectively assist in boar hunts. First of all, they should be relatively small in size to ensure speed, maneuverability, and responsiveness to their handler. Aside from physical fitness, boarhounds need to be independent when hunting, which allows them to follow the game at a distance from the hunter. Boarhounds also need to be stubborn, so that the only footprints they are interested in have been left by the wild boar, not other game. There are diverse breeds used as boarhounds, which differ in size, weight, mobility and temperament. The majority of dogs used as boarhounds belong to three FCI groups (Fédération Cynologique Internationale): terriers, dachshunds, and hounds [1]. Figure 1 presents typical representatives of each group.

Terriers belong to the III group according to the FCI. This group includes 34 breeds, which are divided into four sections: large and medium-sized terriers (e.g., Wire Fox Terrier, German Hunting Terrier, Welsh Terrier and Border Terrier), small-sized terriers (Jack Russell Terrier, West Highland White Terrier and Scottish Terrier), bull-type terriers (Bull Terrier and American Staffordshire Terrier) and toy terriers (Yorkshire Terrier, English Toy Terrier and Australian Silky Terrier) (FCI breeds nomenclature). Most terriers come from Great Britain and are characterized by a hard coat, except for bull-type terriers [2]. They are characterized by strong temperament, courage, hunting passion, and self-confidence [3]. Terriers require a consistent handler who can handle their hard temper. They are mainly used as hunting dogs, guard dogs, and companion dogs [1].

Dachshunds belong to the IV group according to the FCI, which includes nine varieties. There are three types of dachshund: standard dachshund, miniature dachshund, and rabbit dachshund. Each type is further divided based on hair type into: smooth-haired, long-haired and wire-haired [1]. Dachshunds have a distinctive body structure, elongated body, and short legs. Dachshunds are characterized by independence, stubbornness, and problems with subordination. They are used as hunting dogs and companion dogs [2].

Hounds are dogs belonging to the VI group, which includes scent hounds and related breeds. Group VI is divided into three sections. The 1st section includes large-sized hounds (Bloodhound and Grand Griffon Vendéen), medium-sized hounds (Finnish Hound, Polish Hunting Dog and Slovak Kopov) and small-sized hounds (Basset Hound, Beagle and German Hound). The 2nd section includes Alpine Dachsbracke, Bavarian Mountain Scent Hound, and Hanoverian Scent Hounds. The 3rd section includes related dogs, such as Dalmatian and Rhodesian Ridgeback (FCI breeds nomenclature). Hounds are very diverse in size, which means that they are used for hunting game of various sizes. All hounds have good downwind, chasing instincts, and persistence [2].

Hunt trials are the competitions organized in a controlled environment, in which all boarhounds participate and are evaluated in several categories. There are many factors that influence the dog’s performance. The first is, obviously, the breed characteristics that pre-determine the animal’s features. However, in hunting, another important factor is the level of training and contact with the handler. Therefore, in this study, we included dogs that have already started their hunting training. For this reason, hunt trials evaluate not only the breed’s potential but also the quality of the training. Due to the differences in training, we hypothesized that the breed and degree of training had an impact on the results obtained in wild boar competitions. The aim of the study was to determine the factors influencing the hunt trials for boarhounds in a large cohort of hunting dogs.

## 2. Materials and Methods

### 2.1. Hunt Trial Dataset for Boarhounds

The material for the analysis was the results of boarhounds’ competitions and the data contained in the “Catalog of hunting dog work trials and competitions” in 2005–2015. The catalog was developed by the Polish Hunting Association (PHA). The dataset included boarhounds, aged at least 9 months and older (without an upper age limit), which have undergone training and participated in at least one hunt trial. The hunt trial for boarhounds consists of ten different competitions, including: scent (nose), searching, courage and sharpness, voice on trail, voice when game in sight, correctness of attack, persistence, obedience, tracking on the lead and announcing/barking. The description of the competition and the points for each category is given in Table 1. Ratings for each competition are awarded by the Judges of the Kennel Club in Poland. The rating scale is between 0 (lowest score) and 4 (highest score). The final score is the sum of the obtained rating and the weights corresponding to the given competition. Weather and environmental conditions were not included in the scoring algorithm. The complete dataset analyzed in this study is available in Supplementary File S1.

### 2.2. Statistical Analysis

The data were checked for compliance with normal distribution using the Kolmogorov–Smirnov test. The lack of a normal distribution resulted in the use of non-parametric analysis of variance. The influence of gender on the values of the grades achieved during hunt trials for boarhounds was checked using Mann–Whitney U statistics, while the influence of age, breed group and breed was assessed using the Kruskal–Wallis test with Dunn’s correction for multiple comparisons. The Mann–Whitney U statistics and Kruskal–Wallis H test with Dunn’s correction for multiple comparisons were used for variables with 2 groups and variables with more than 2 groups, respectively. For the analysis of the influence of age, all animals were included and divided into six age groups: I (9–24 months of age), II (25–48 months of age), III (49–72 months of age), IV (73–96 months of age), V (97–120 months of age), and VI (121 and above months of age). The influence of three breed groups according to the FCI was analyzed: III terriers, IV dachshunds and VI hounds and related breeds. For the analysis of the influence of breed on the value of the evaluation obtained for a given competition, eight breeds were selected, the number of which exceeded 50 individuals. The calculations were made in the SAS 9.4 software Copyright (c) 2002–2012 by SAS Institute Inc., Cary, NC, USA and in the MedCalc Statistical Software version 19.2.6 (MedCalc Software bv, Ostend, Belgium; https://www.medcalc.org; 2020) (accessed on 5 February 2021).

## 3. Results

### 3.1. Dog Breeds and General Results

**Figure 2:**
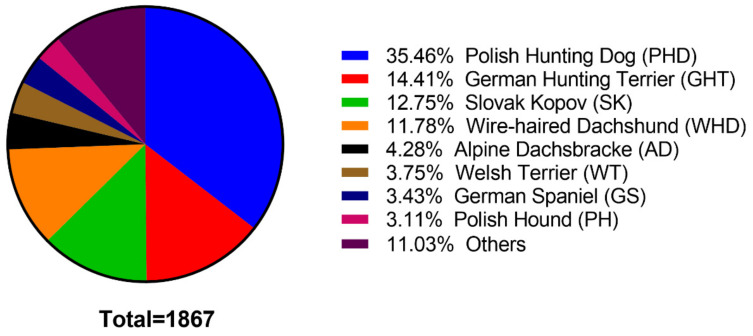
Breed distribution of the boarhound population.

The complete dataset included 1867 individuals belonging to 39 dog breeds. Figure 2 presents the breed distribution analyzed in this study. The most numerous breeds were Polish Hunting Dog (662 individuals), German Hunting Terrier (269 individuals), Slovak Kopov (238 individuals) and Wire-haired Dachshund (220 individuals). All boarhound breeds analyzed in this study have been grouped into three breed groups, i.e., (1) hounds (*n* = 1109), (2) terriers (*n* = 373), and (3) dachshunds (*n* = 233). All animals (*n* = 1867) were included in variance analysis, in which sex and age were factors influencing the results of the hunt trials. Data reduction was applied for variance analysis in which breed or breed group were factors. Breeds represented by a small number of individuals (<50), e.g., English Springer Spaniel, Hanoverian Scent Hound, or German Short-haired Pointing Dog, were excluded from the analysis of the influence of the breed (*n* = 1661) on the hunt trials. In the breed group, there were 152 dogs removed from the database (*n* = 1715). The reason for data reduction in this case was to sort out breeds that were classified as flushing dog, pointing dog, spitz and primitive types.

The Table 2. present results of the hunt trials for boarhounds. In hunt trials for boarhounds, dogs achieved scores from 0 to 180 points. The mean score was 116.74. The boarhounds obtained the highest results in obedience (3.69), scent (nose) (3.54), and searching (3.47), while the lowest results were scored in announcing/barking (0.24), and tracking on the lead (2.76).

### 3.2. Sex Effect

Table 3 presents the effects of sex on the results of the hunt trials in boarhounds. A total of 1104 dogs and 763 bitches were evaluated altogether. Dogs scored numerically higher than bitches in every competition. Dogs also scored numerically higher than the average for the entire study population (significance was not tested). Highly significant differences between sexes occurred in the total score and the competitions: scent (nose), searching, courage and sharpness, voice on trail, voice when game in sight, correctness of attack, and persistence (*p* < 0.01). No statistical significance was found in obedience, tracking on the lead, and announcing/barking (*p* > 0.05).

### 3.3. Age Effect

All dogs, i.e., 1867 animals, were used to analyze the influence of age, and the results are presented in Table 3. The dogs were divided into six age groups. The most numerous group were dogs from group II (615 individuals), and the least numerous group was group I (146 individuals). There was a constant increase in the scores received for each competition with age. Scores equal to or above the average occurred in group III and older in the total score and in the following competitions: scent (nose) (*p* < 0.05), searching, courage and sharpness, correctness of attack, obedience, tracking on the lead and announcing/barking (*p* < 0.01). In all categories, the older dogs achieved higher results than the younger ones, especially in the obedience, voice and persistence competitions (*p* < 0.01). The smallest differences occurred in the scent (nose) and announcing/barking competition and involved the youngest (groups 1 and 2) and the oldest dogs (group 6) (*p* < 0.01).

### 3.4. Breed Group Effect

Table 4 presents the effects of breed group on the results of the hunt trials in boarhounds. The cohort size for the breed group (*n* = 1715) included only typical boarhounds (i.e., hounds, terriers, and dachshunds), which were predominant groups in the dataset. The remaining groups belonged to pointing dogs, flushing dogs, and spitz and primitive types, which were numerically underrepresented and therefore omitted from this analysis. There were three main groups in boarhound hunt trials: hounds (1109 individuals), terriers (373 individuals), and dachshunds (233 individuals). The most numerous groups were hounds, the leading representative of which was the Polish Hunting Dog, and the least numerous were the dachshunds with the Wire-haired Dachshund as the typical representative. The mean of the total score among the three breed groups was 117.40, and it was the highest mean among all the scores analyzed. The boarhounds classified into three breed groups (based on FCI classification) obtained the highest results for obedience (3.69) and scent (nose) (3.55), and the lowest for announcing/barking (0.24). The best total score was achieved by the dachshund dogs (119.96), and the lowest by the terriers (114.26) (*p* < 0.01). Dachshunds scored numerically higher than or equal to almost all competitions except scent (nose), obedience and announcing/barking. There were no differences between the breed groups in scent (nose), voice on trail, correctness of attack and obedience (*p* > 0.05).

### 3.5. Breed Effect

Table 5 presents the effects of breed on the results of the hunt trials in boarhounds. The effect of breed was analyzed in eight breeds (1661 individuals), with a total of more than 50 individuals. The most numerous breed was Polish Hunting Dog (662 individuals), and the least numerous was Polish Hound (58 individuals). The mean for the total score among the analyzed breeds was 117.27 points. Boarhounds achieved the highest results in the obedience competition (3.71) and the lowest in the announcing/barking competition (0.24). Highly significant differences were found in the overall score between breeds (*p* < 0.01). The Welsh Terrier and Polish Hound differed from the Alpine Dachsbracke, Polish Hunting Dog, Wire-haired Dachshund, German Hunting Terrier and Slovak Kopov. No differences between breeds occurred in the tracking on the lead competition (*p* > 0.05). The smallest differences were in the obedience and search competition. By contrast, the greatest differences between breeds were in the courage and sharpness competition. The Polish Hound achieved the lowest score among all the competitions. The Alpine Dachsbracke scored numerically the highest in most of the competitions, except persistence, obedience, and announcing/barking.

## 4. Discussion

### 4.1. Overview

In this study, we have analyzed a large population of boarhounds subjected to routine and standardized hunt trials. Such trials are an important part of the selection process of the hunting dogs. Hunting dogs should be characterized by intelligence, obedience, courage and trainability. Training a hunting dog is based on their hunting potential and instincts. The strength of the hunting instinct depends on the breed characteristic and individual properties. The hunt trials are designed to compare different individuals within the same dogs’ type (in this case, boarhounds). The boarhounds are scored in various categories that reflect the skills they should practice in a typical hunt for wild boar. A single individual can take the hunt trial several times, so it is also possible to evaluate the effects of training and experience that the dog gains over time. The analysis of the scores showed that all factors (i.e., sex, age, and breed) influenced the final performance of the dogs. Dogs achieved numerically higher scores than bitches. Older dogs achieved higher scores compared to the younger dogs. Dachshunds were the best among the breed groups. The highest results in most of the competitions were obtained by the Alpine Dachsbracke, and the lowest by the Polish Hound.

### 4.2. Sex Effect

Highly significant differences between the sexes occurred in the hunt trials for boarhounds. Dog boarhounds outperformed bitches in the total score and all individual competitions. These results were significant for the entire population of the boarhounds, but even more prominent when the individual breeds were taken into account in the analyses (data not shown). The largest disproportion in the performance during the hunt trials between the sexes of the boarhounds was in Alpine Dachsbracke and the Slovak Kopov, which were the two best performing dog breeds. This shows that the merit of the breed is mainly contributed from the male side. Those differences were in line with the general characteristics associated with sex in the canine; the dogs are able to move faster than the bitches and they are capable of greater effort [1]. Studies conducted on different breeds demonstrated that dogs tend to express greater boldness and courage than bitches, which sometimes turns into aggression [4].

In the junior hunting test for pointing dogs, a sex effect was demonstrated for the total score, speed, and swimming. Dogs achieved better scores than bitches [5]. The hunting behavior test on the breed Flat-coated Retriever showed sex differences in competitions reaction on shot, reaction when throwing the game, cooperation and waiting passively in group. Dogs scored higher than bitches except for cooperation, but dogs were also more extent [6]. In the analysis of the survey about dog behavior, the most common breed, English Springer Spaniel, showed no difference between the sexes with the exception of the level of aggression towards other dogs. The dogs achieved significantly higher levels than the bitches [7]. In behavioral tests analyzed by van der Waaij et al. (2008), bitches scored lower than dogs in the German Shepherd Dog breed in the case of sharpness, and in Labrador Retriever co-operation and gun shyness. In the case of other features, such as defense drive and nerve stability, they were also greater in both breeds, except for temperament and affability [8].

Bitches are generally easier to style and less prone to dominance [1]. These characteristics of bitches may be the result of a higher adaptive intelligence as determined by the Intelligence Quotient test, during which bitches scored higher on most tasks [9]. Various studies show that social skills are more associated with bitches [4]. For example, in studies of most popular dog breeds in Italy, they found significant sex effects for all traits except playfulness. Bitches scored higher in demand for affection, ease of housetraining and obedience training. Dogs achieve higher score in destructiveness, general activity, excessive barking, snapping at children, watchdog behavior, excitability, territorial defense, dominance over the owner and aggressiveness towards other dogs [10].

In the years 1995–1996, a test was carried out on spitz hunting dogs (Norwegian Elkhound), scent dogs (Hare Hunting Dog) and pointing dogs (English Setter). The test consisted of checking the dog’s behavior towards sheep. The study found no effect of sex on chasing and attacking sheep [11]. C-BARQ on Labrador Retriever dogs showed a significant sex effect only on most traits, especially for traits such as attention, noise fear, separation anxiety, attachment, chasing, energy level, non-social fear, separation-related behavior and stranger-directed aggression [12].

### 4.3. Age Effect

The age group significantly influenced almost all the competition, except for the wind. The scores in the hunt trials increased with age. The main reason that explains such results is probably more training and experience that the dogs gain with time, which makes them more mature and focused. A more pronounced influence of the age on the results of the hunting behavior test was found by Lindberg et al. (2004) performed on Flat-Coated Retrievers. They found a significant impact of age on the interest and efficiency in searching and retrieving a test object from water as well as retrieving and grip [6]. In the junior hunting test for pointing dogs, the effect of age was apparent (older dogs performed better) in the swimming competition, which required the young dog to enter the water on demand [5].

Depending on the breed, different physical and psychological characteristics of the dogs change with age. Kubinyi showed a relationship between age and calmness; [13] the older dogs were calmer, but younger dogs were more trainable, social and bold. Svartberg (2002) also found a negative correlation between age and boldness [14]. In studies of the behavior of spitz and primitive types of dogs, scent dogs and pointing dogs have been found to influence the frequency of attacks on sheep. The older the dogs, the less often they attacked the sheep [11]. In studies of dogs on the basis of a questionnaire carried out on purebred dogs (including Hungarian Vizsla and German Shepherd Dog) and mongrels, the influence of age on activity was shown. Younger dogs were more active [15]. In Swedish behavioral tests, age played a significant role in the achieved results. As the age increased, the dogs scored higher. The age effect was more pronounced with the German Shepherd Dog than with Labrador Retrievers [8]. In the analysis of the responses to C-BARQ of Labrador Retriever dogs, age was found to have an effect only on fetching and energy levels [12].

Age is obviously related to comprehension and learning ability, which was proven in research on using the pointing gesture or marker as a cue, performed on three groups of dogs: primitive (Akita Inu, Alaskan Malamute), hunting/herding dogs (Australian Shepherd), and molossoid (Boxer, Bull Terrier). Regardless of their age, all puppies read the indication gestures in a correct manner, but there were differences in the reading of the marker; the younger dogs coped worse [16].

### 4.4. Breed Group Effect

The breed group that outperformed others in the general score of hunt trials for boarhounds was dachshunds, before hounds and terriers. Dachshunds are brave, but also very demanding because of their independent character and stubborn behavior. That breed group is also very courageous and clever [17]. The highest scores for dachshunds may be attributed to the smaller dogs being better at individual hunts than hounds, and they are easier to control than terriers [1].

Hounds had a higher score in the scent (nose) competition, obedience and announcing/barking than dachshunds and terriers. The best results in the scent (nose) competition may result from the fact that this group has a very good lower wind. The highest score in announcing/barking competition can be the effect of a very loud voice. The highest results in obedience may have been due to the fact that terriers and dachshunds are dogs of a more difficult character [1]. Terriers had the lowest results in almost all competitions except persistence, courage and sharpness. The lowest results in terriers may be because of obedience problems often attributed to this breed group, and therefore they need an experienced handler. However, they have great hunting instincts, strong character, passion, and courage, which is reflected in the results [1].

In the study of dog behavior towards sheep, the spitz and primitive type dogs were of the highest interest compared to pointing dogs and scent dogs [11]. The influence of the breed group on the characteristics of dogs was shown in the studies on trainability and boldness results. The best results for trainability were achieved by herding dogs, then hounds, working dogs, toy dogs and non-sporting dogs, while for boldness, terriers achieved higher scores than hounds and herding dogs [18]. Behavior analysis based on the dogs’ personality questionnaire showed that terriers turned out to be more sociable than mongrels. Sheep and cattle dogs and terriers were more active dogs than sighthounds. Retriever dogs were less trainable than mongrels [15]. In studies using the Canine Behavioral Assessment and Research Questionnaire, the influence of the age group on boldness was examined. Terriers achieved a moderate score of boldness [19]. Terriers showed the greatest curiosity and lack of fear [20].

### 4.5. Breed Effect

The Alpine Dachsbracke belongs to the hound group and is the most versatile hunting dog in this group. The high score in the scent nose competition of Alpine Dachsbracke is probably due to the fact that it is a breed with excellent breath. A characteristic feature is the use of voice in the pursuit of animals, which was also reflected in the results of the announcing/barking competition, because it achieved the second result after the Polish Hunting dog. They are also very sharp against game, achieving the highest score in the courage and sharpness competition. Alpine Dachsbracke is a breed of dog that requires training due to their high individuality [21]. Breeders should be careful about inbreeding in this breed as in countries where there is a small population there may be high inbreeding in the breed. In Poland, the analysis of four-generation inbreeding showed that in some individuals it was as high as 25% [22].

The most common Polish breed of boarhounds is the Polish Hunting Dog, which did not perform too well in the hunt trial. The Polish Hunting Dog is energetic and intelligent. The very average results of the Polish Hunting Dog could have been caused by mistakes made during the training. The Polish Hunting Dog is a breed of dog that is difficult to train and has a high temperament, so it can be problematic especially in the case of young inexperienced keepers [23]. The level of inbreeding in this breed is at a good level [24].

The lowest results for this competition, as for others, were for the Polish Hound, probably due to the fact that in this breed the hunting instinct is muted [1]. The Polish Hound is a breed that is currently used as a companion dog because of their average temperament and balanced character. It is a dog with high sensitivity to stimuli; therefore, socialization is very important in its case [25]. Polish Hound breeding should pay attention to the selection of individuals for breeding because, as shown by inbreeding studies in 1960–2004, the level of inbreeding of some individuals was up to 37% [26].

In the competition of courage and sharpness, the Welsh Terrier scored one of the lowest results, in contrast to the tests carried out on various utility types, including terriers [27]. In the test of the North American Versatile Hunting Dog Association, the highest score was achieved by the German Short-haired Pointer (3.32) and the Pudelpointer (3.25), and the lowest by the Griffon (2.85) [28]. In the analysis of the working dog competitions, the higher results were achieved by the German Shepherds in the shyness–boldness dimension. Beagle and Labrador Retriever scored higher in sociability than German Shephard Dog and Jack Russell Terrier. However, the Labrador Retriever and Jack Russell Terrier scored higher in boldness [20]. Trainability was analyzed on the basis of C-BARQ in 11 breeds (including Labrador Retriever, English Springer Spaniel, Dachshund and Basset Hound). The dogs performing the highest were the Labrador Retriever and the lowest—the Basset Hound [29]. In the junior hunting test for pointing dogs, the breed effect was shown for all competitions. The highest results were achieved by German Short-haired and Wire-haired Pointer, and the lowest by English and Irish Setters [5].

## 5. Conclusions

In this study, we assessed the results of the hunt trials in boarhounds. The highest scores were obtained by older and mentally more mature individuals who were better prepared for hunt trials due to longer training and greater hunting experience. The dogs showed better results than the bitches during the hunt trials, which shows their bolder character, while in the competitions based on interaction with handlers both sexes performed on a similar level. The best performing breed was Alpine Dachsbracke, and the breed type—dachshunds. The results can be used to follow up the breeding and training programs of hunting dogs.

## Figures and Tables

**Table 1:** Description of traits and scoring system included in hunt trial for boarhounds.

Trait	Score	Weight	Description
Scent (Nose)	0–4	4	Catching and following the scent of the game
Searching	0–4	6	The depth, width and speed of the dog’s gait
Courage and sharpness	0–4	5	The distance and its behavior towards the boar while attacking
Voice on trail	0–4	4	The rhythm and frequency of the voice produced during the search for and attack on the boar
Voice when game in sight	0–4	4	The correctness of the voice when finding the trail or the game
Correctness of the attack	0–4	5	The dog should attack the boar from the front or from the rear, keeping a sufficient and safe distance
Persistence	0–4	5	The dog’s persistence is assessed when herding the boar towards the handler, and each time the dog leaves and moves away from the boar, the score is lowered.
Obedience	0–4	4	The correctness of the dog’s walking on a leash at the leg and the dog’s coming on call are assessed, the assessment is carried out before other competitions
Tracking on the lead	0–4	2	The ability to track the traces of boar or pig blood previously left by the organizers by a dog kept on a 6-meter-long lead
Announcing/barking	0–4	2	Additional competition, aims at notifying the handler about finding the game by approaching the game and barking until the handler arrives

**Table 2:** Descriptive statistics of the results of the hunt trials for boarhounds.

Traits	Mean	SD	Min.	Max.	Sex	Age	Breed Group	Breed
Total score	116.74	27.08	0	180	**	**	**	**
Scent (Nose)	3.54	0.84	0	4	**	*	ns	**
Searching	3.47	0.86	0	4	**	**	*	**
Courage and sharpness	3.15	1.02	0	4	**	**	**	**
Voice on trail	3.37	0.95	0	4	**	**	ns	**
Voice when game in sight	3.28	1.03	0	4	**	**	*	**
Correctness of the attack	3.25	1.02	0	4	**	**	ns	**
Persistence	3.09	1.08	0	4	**	**	**	**
Obedience	3.69	0.61	0	4	ns	**	ns	*
Tracking on the lead	2.76	1.15	0	4	ns	**	*	ns
Announcing/barking	0.24	0.83	0	4	ns	*	**	**

Significance thresholds: * *p* < 0.05; ** *p* < 0.01; ns *p* > 0.05.

**Table 3:** The impact of sex and age on the results of the hunt trials for boarhounds.

Traits	Mean	Sex		*p*	Age						*p*
		Bitch	Dog		I	II	III	IV	V	VI	
N	1867	763	1104		146	615	440	269	165	232	
N Bitch/Dog					68/78	286/329	172/268	95/174	58/107	84/148	
Total score	116.74	112.98 ^A^	119.35 ^B^	**	107.44 ^CDEF^	113.73 ^CEF^	117.25 ^ABF^	117.86	121.99	124.51	**
Scent (Nose)	3.54	3.45 ^A^	3.61 ^B^	**	3.36 ^F^	3.53	3.54	3.57	3.53	3.67 ^A^	*
Searching	3.47	3.37 ^A^	3.54 ^B^	**	3.30	3.40 ^F^	3.49	3.51	3.49	3.62 ^B^	**
Courage and sharpness	3.15	3.01 ^A^	3.24 ^B^	**	2.88 ^EF^	3.06 ^F^	3.17	3.15	3.30 ^A^	3.63 ^AB^	**
Voice on trail	3.37	3.26 ^A^	3.45 ^B^	**	3.11 ^CDEF^	3.28 ^EF^	3.35 ^AF^	3.45 ^A^	3.52 ^AB^	3.62 ^ABC^	**
Voice when game in sight	3.28	3.17 ^A^	3.36 ^B^	**	3.01 ^DEF^	3.19 ^F^	3.24 ^F^	3.35 ^A^	3.42 ^A^	3.58 ^ABC^	**
Correctness of the attack	3.25	3.12 ^A^	3.33 ^B^	**	2.93 ^CEF^	3.16 ^EF^	3.29 ^A^	3.25	3.40 ^AB^	3.47 ^AB^	**
Persistence	3.09	2.93 ^A^	3.19 ^B^	**	2.74 ^CDEF^	2.97 ^EF^	3.08 ^AEF^	3.08 ^AEF^	3.36 ^ABCD^	3.43 ^ABCD^	**
Obedience	3.69	3.67	3.70	ns	3.49 ^BCDF^	3.68 ^A^	3.71 ^A^	3.74 ^A^	3.66	3.73 ^A^	**
Tracking on the lead	2.76	2.69	2.81	ns	2.56 ^EF^	2.65 ^EF^	2.79	2.74	2.97 ^AB^	3.00 ^AB^	**
Announcing/barking	0.24	0.20	0.26	ns	0.14	0.16 ^E^	0.25	0.23	0.46 ^B^	0.32	**

Significance thresholds: * Significance thresholds: * *p* < 0.05; ** *p* < 0.001; ns *p* > 0.05; Numbers in superscripts of the mean results in the age groups indicate the differences to other age groups (A—group I, B—group II, C—group III, D—group IV, E—group V and F—group VI).

**Table 4:** The impact of breed groups on the results of the hunt trials for boarhounds.

Traits	Mean	Breed Groups According to FCI			*p*
		Hounds	Dachshunds	Terriers	
N	1715	1111	231	373	
N_Bitch_		457	90	155	
N_Dog_		654	141	218	
Total score	117.40	117.92 ^C^	119.96 ^C^	114.26 ^AB^	**
Scent (Nose)	3.55	3.57	3.54	3.50	ns
Searching	3.48	3.50	3.54	3.37	*
Courage and sharpness	3.17	3.13 ^B^	3.34 ^A^	3.18	**
Voice on trail	3.39	3.40	3.49	3.30	ns
Voice when game in sight	3.31	3.31	3.46 ^C^	3.21 ^B^	*
Correctness of the attack	3.26	3.26	3.39	3.21	ns
Persistence	3.11	3.07 ^B^	3.31 ^A^	3.10	**
Obedience	3.69	3.72	3.67	3.62	ns
Tracking on the lead	2.78	2.82 ^C^	2.82	2.64 ^A^	*
Announcing/barking	0.24	0.34 ^BC^	0.11 ^A^	0.05 ^A^	**

Significance thresholds: * *p* < 0.05: ** *p* < 0.01: ns *p* > 0.05: Numbers in superscripts of the mean result in breed groups indicate the differences to the other breed groups (A—Hounds, B—Terriers, C—Dachshunds).

**Table 5:** The impact of breed on the results of the hunt trials for boarhounds.

Traits	Mean	Breeds								*p*
		1. PHD	2. GHT	3. SK	4. WHD	5. AD	6. WT	7. GS	8. PH	
N	1661	662	269	238	220	80	70	64	58	
N_Bitch_	672	269	115	94	84	32	27	23	28	
N_Dog_	989	393	154	144	136	48	43	41	30	
Total score	117.27	117.61 ^FH^	117.143 ^FH^	120.06 ^FGH^	120.03 ^FH^	125.26 ^FGH^	108.77 ^ABCDE^	106.01 ^CE^	104.12 ^ABCDE^	**
Scent (Nose)	3.55	3.56 ^H^	3.55 ^H^	3.63 ^H^	3.55 ^H^	3.75 ^H^	4.49	3.30	3.10 ^ABCDE^	**
Searching	3.47	3.50 ^H^	3.39	3.56 ^H^	3.54 ^H^	3.64 ^H^	3.45	3.14	3.03 ^ACDE^	**
Courage and sharpness	3.17	3.11 ^BDE^	3.32 ^AFGH^	3.23	3.40 ^AFGH^	3.46 ^AFGH^	2.83	2.75 ^BDE^	2.73 ^BCDE^	**
Voice on trail	3.39	3.37 ^CE^	3.39 ^GH^	3.53 ^AGH^	3.50 ^GH^	3.68 ^AFGH^	3.21 ^E^	3.03 ^BCDE^	2.96 ^BCDE^	**
Voice when game in sight	3.29	3.26 ^E^	3.26 ^FG^	3.39 ^FGH^	3.46 ^AGH^	3.61 ^AFGH^	2.99 ^BCDE^	2.85 ^BCDE^	2.94 ^CDE^	**
Correctness of the attack	3.26	3.24	3.34 ^FH^	3.35 ^FH^	3.39 ^FH^	3.42 ^FH^	2.86 ^BCDE^	2.84	2.94 ^BCDE^	**
Persistence	3.09	2.99 ^BCD^	3.18 ^AGH^	3.27 ^AGH^	3.31 ^AGH^	3.30 ^GH^	2.95	2.50 ^BCDE^	2.70 ^BCDE^	**
Obedience	3.71	3.76	3.61	3.70	3.68	3.80	3.61	3.83	3.52	*
Tracking on the lead	2.79	2.82	2.72	2.81	2.83	3.04	2.47	2.63	2.67	ns
Announcing/barking	0.24	0.41 ^BCDF^	0.06 ^A^	0.19 ^A^	0.11 ^A^	0.31	0.00 ^A^	0.25	0.07	**

Significance thresholds: * *p* < 0.05: ** *p* < 0.01: ns *p* > 0.05: Numbers in superscript of the mean results in the breed indicate the difference to other breeds (A—PHD Polish Hunting Dog, B—GHT German Hunting Terrier, C—SK Slovak Kopov, D—WHD Wire-haired Dachshund, E—AD Alpine Dachsbracke, F—WT Welsh Terrier, G—GS German Spaniel H—PH Polish Hound).

## Data Availability

The data are available in Supplementary File S1.

## Supplementary Materials

The following are available online at https://www.mdpi.com/article/10.3390/ani11061661/s1, Supplementary File S1.
